# Editorial: Molecular systematics and phylogeography of tropical and subtropical biodiversity

**DOI:** 10.3389/fgene.2023.1345239

**Published:** 2023-12-13

**Authors:** Victor Hugo Valiati, Fernando Lopes

**Affiliations:** ^1^ Laboratório de Genética e Biologia Molecular, Programa de Pós-Graduação em Biologia, Centro de Ciências da Saúde, Universidade do Vale do Rio dos Sinos, São Leopoldo, Brazil; ^2^ Finnish Museum of Natural History, University of Helsinki, Helsinki, Finland

**Keywords:** global south, inequalites, funding, disparity, genomics

Scientific publishing reveals a troubling disparity that often goes unnoticed. Researchers from the Global South (social perspective), where the largest biodiversity richness is concentrated (Cardoso et al., 2011; Raven et al., 2020), encounter severe obstacles that limit their ability to contribute meaningfully to the scientific community (Nakamura et al., 2023). These hindrances, deeply rooted in socio-economic and structural issues, cast a long shadow over our endeavors, promoting an ever-growing inequality between both regions (Castro Torres and Alburez-Gutierrez, 2022). Another striking challenge is the shortage of resources that institutions in the Global South constantly face, such as inadequate funding and the lack of cutting-edge facilities, reducing the breadth and caliber of their scientific pursuits. This scenario stems from the complete lack of long-term science and technology policies with well-established goals. Economic instability and governmental lack of interest in science have a profound effect on studies that seek to recognize biodiversity and conservation in unprivileged regions (Young, 2005; Lund et al., 2023). For example, Brazil, one of the most megadiverse countries, constantly faces budget cuts in fundamental areas (e.g., science), as recently occurred. The former Brazilian government (Bolsonaro’s presidency term, 2018–2022) approved restrictions to the federal budget for science and education by more than 90% (Kowaltowski, 2021), affecting ongoing projects and compromising scientific productivity at all academic levels.

Moreover, access to critical scientific literature remains an arduous task. Exorbitant subscription costs for top-ranked journals and databases raise an almost impenetrable barrier, depriving these researchers of the essential foundation upon which to build their high-quality work (Vervoort et al., 2021; Kwon, 2022). These expensive article processing fees (APCs) are not covered by funding. In addition, in countries with fragile economies, such as in many countries from the Global South, there is a further aggravating factor: unfavorable currency conversion rates (Lund et al., 2023).

This problem can be exacerbated by funding disparity and the predominance of English-language publications, which can exclude valuable research conducted in other languages, constraining a more diverse cross-cultural pollination of ideas (Amano et al., 2023). However, these challenges sometimes extend beyond finances and language barriers, being affected by political and economic instability, which further disrupts research activities and their communication (Young, 2005).

Addressing these rooted entrenched issues demands a global effort, and the recognition of such obstacles is the first step to be taken. To address these disadvantages, collective efforts are required, including supporting open-access initiatives, fostering international collaborations, and recognizing the importance of diversity in advancing global scientific research (Grahe et al., 2020). Taking all of it into account, the promotion of these valuable aspects of science was the cornerstone of this Research Topic. Here, we contributed to “remove one brick of the wall” by focusing on shortening physical and financial distances and diminishing the barriers in the field of genomics of molecular systematics and phylogeography.

Due to limited financial resources as a result of the aforementioned, the use of genomic data to understand biodiversity, species and population relationships, and the evolution of non-model organisms is usually out of reach to local researchers, leading to a gap in evolutionary knowledge between North and South (Helmy et al., 2016). In light of this, the focus of the current Research Topic was to promote the use of genomics for molecular systematics and phylogeographical studies of tropical and subtropical biodiversity from developing countries as featured below.

Lopes et al. showed that, although mammal feces are undeniably important, the role of bird and reptile dung (=Sauropsida) may have been greatly underestimated in the evolution of dung beetles (Coleoptera: Scarabaeinae). Using amplicon-metagenomics they identified host species from fecal pellets associated with a meta-analysis of available records, associating dung beetles from insular environments also to sauropsids. The authors reported that generalist species of dung beetles from Mauritius and Madagascar are strongly attracted to sauropsid dung. Furthermore, the results provide evidence that there is no constraint that the evolution of coprophagy in beetles could have been triggered by dinosaur dung.

Lyra et al. used complete mitogenomes and genome-wide single nucleotide polymorphism data to infer the evolutionary history of an endemic group of grass frogs of the genus *Ptychadena* from the Ethiopian Highlands, one of the main centers of endemism and biodiversity in continental Africa (Williams et al., 2004; Fashing et al., 2022). They used these molecular markers to gain a better understanding of how the characteristics of the landscape influenced the population structure and dispersal of these frogs over time and space. The authors were able to suggest a well-founded biogeographical hypothesis based on a dated phylogenetic tree and pointed out that the diversification of the species of grass frog results from a combination of factors involving the geological history of the Ethiopian highlands and environmental changes due to climatic oscillations.

A different approach is presented by Fonseca et al. by developing a bait kit targeting 762 nuclear genes, including 329 genes selected specifically for the Bignoniaceae; 348 genes obtained from the Angiosperms353 (Johnson et al., 2018), with baits designed specifically for the family; and 85 low-copy genes of known function. According to the authors, the great diversity and high homoplasy of the reproductive morphological structures of Bignoniaceae have resulted in considerable taxonomic problems, despite the most recent phylogenetic hypotheses and lineages of the family and genera (Lohmann, 2006; Grose and Olmstead, 2007; Olmstead et al., 2009). The authors’ strategy allowed the selection of 677 loci of orthologous nuclear protein-coding genes using the Hyb-Seq Pipeline (Weitemier et al., 2014), except probe design based on the *Handroanthus impetiginosus* genome combined with the three transcriptomes. This set of loci was used for phylogenetic analyses and resulted in a well-founded phylogenetic hypothesis of Bignoniaceae.

Battaglia et al. provided new findings on the global phylogeny and phylogeography of the worldwide spread of *Aedes albopictus* revealed using mitochondrial genomes. *Aedes albopictus* is the vector of a considerable number of arboviruses that cause various diseases (Kraemer et al., 2019) in many countries, and its recent expansion is a major concern for global public health (Swan et al., 2022; Laporta et al., 2023). The species is characterized by three divergent haplogroups (A1, A2, and A3), with only haplogroup A1 recognized as being associated with its recent spread. In the study, the 76-tiger mosquito mitogenomes revealed new branches and sub-branches within this important A1 haplogroup in terms of the invasion history of this species. At the same time, the authors demonstrated that clonal and subclonal founder events have strongly influenced the current distribution of mitogenome variation. In particular, possible starting points for the two main clades within haplogroup A1 were pointed out. In this scenario, A1 would have spread mainly along temperate areas of Japan and China, and A1b would have emerged and disseminated mainly in tropical areas of Southern Asia, with subsequent expansion into the tropical areas of Africa (Cameroon). It also reaches non-tropical areas with very similar climatic conditions to subtropical regions, such as Greece and Florida.

The study by Snead et al. evaluated the genetic structure of *Kryptolebias marmoratus*, a species of intertidal fish widely distributed in the highly fragmented mangrove forests of the New World in Central America, the Caribbean, the Bahamas, and Florida. All in all, the patterns of gene flow that emerged from this study were influenced by dispersal distance and/or environmental factors that influence dispersal between populations (Slatkin, 1987). Authors’ view, mangrove patches are biological islands with marked differences in abiotic and biotic conditions compared to the adjacent habitat and this landscape may have influenced gene flow and how genetic variability would be distributed spatially. The study combined more than 1,000 genetic samples and the genotyping of 32 microsatellites with spatial analysis and biophysical modeling covering 10 years of information on ocean currents to assess the impact of wind and currents on the genetic distribution of these fish. Analysis of molecular variance indicated a significant population genetic structure at different levels (regional, local, population, and individual), indicating that mangrove rivulus fish show genetic differentiation on large and fine scales. The integration of the robust genetic database and biophysical modeling has shown that ocean currents and the distance between populations influence the passive dispersal and gene flow patterns hypothesized by the authors. According to migration estimates, some asymmetries in the gene flow appear to be mediated by ocean currents and distances.
